# Chromosome X-wide association study in case control studies of pathologically confirmed Alzheimer’s disease in a European population

**DOI:** 10.1038/s41398-024-03058-9

**Published:** 2024-09-04

**Authors:** Emily Simmonds, Ganna Leonenko, Umran Yaman, Eftychia Bellou, Amanda Myers, Kevin Morgan, Keeley Brookes, John Hardy, Dervis Salih, Valentina Escott-Price

**Affiliations:** 1 Dementia Research Institute, Cardiff University, Cardiff, UK; 2 Institute of Neurology, University College London, London, UK; 3 Department of Cell Biology, University of Miami, Miller School of Medicine, Miami, FL USA; 4 University of Nottingham, Nottingham, UK; 5 Biosciences, School of Science and Technology, Nottingham Trent University, Nottingham, UK; 6 Division of Psychological Medicine and Clinical Neurosciences, School of Medicine, Cardiff University, Cardiff, UK

**Keywords:** Genetics, Diseases

## Abstract

Although there are several genome-wide association studies available which highlight genetic variants associated with Alzheimer’s disease (AD), often the X chromosome is excluded from the analysis. We conducted an X-chromosome-wide association study (XWAS) in three independent studies with a pathologically confirmed phenotype (total 1970 cases and 1113 controls). The XWAS was performed in males and females separately, and these results were then meta-analysed. Four suggestively associated genes were identified which may be of potential interest for further study in AD, these are *DDX53* (rs12006935, OR = 0.52, *p* = 6.9e-05), *IL1RAPL1* (rs6628450, OR = 0.36, *p* = 4.2e-05; rs137983810, OR = 0.52, *p* = 0.0003), *TBX22* (rs5913102, OR = 0.74, *p* = 0.0003) and *SH3BGRL* (rs186553004, OR = 0.35, *p* = 0.0005; rs113157993, OR = 0.52, *p* = 0.0003), which replicate across at least two studies. The SNP rs5913102 in *TBX22* achieves chromosome-wide significance in meta-analysed data. *DDX53* shows highest expression in astrocytes, *IL1RAPL1* is most highly expressed in oligodendrocytes and neurons and *SH3BGRL* is most highly expressed in microglia. We have also identified SNPs in the *NXF5* gene at chromosome-wide significance in females (rs5944989, OR = 0.62, *p* = 1.1e-05) but not in males (*p* = 0.83). The discovery of relevant AD associated genes on the X chromosome may identify AD risk differences and similarities based on sex and lead to the development of sex-stratified therapeutics.

## Introduction

Alzheimer’s Disease (AD) is the most common form of dementia accounting for between 60 and 80% of dementia cases [1]. The prevalence of AD in women is higher than that in men [1, 2]. This may be because women tend to live longer than men [3, 4]. However, some studies have suggested that women over 80 years may be more likely to have AD than men of the same age [5]. The effect of sex has a varying impact on AD over the course of the disease [6]. The duration of ovarian hormone exposure protects against dementia [7], i.e., shorter oestrogen exposure in women was associated with higher dementia risk in the UK Biobank [8]. A number of genetic loci have sex-specific effects on AD [6], for example, the risk of AD associated with a given *APOE* genotype changes with sex [9]. The presentation of disease can differ by the sex of the individual with AD, highlighting the impact of sex on disease heterogeneity. Men and women demonstrate different cognitive and psychiatric symptoms. After diagnosis of either MCI or AD, women show faster cognitive decline. For those with mild cognitive impairment (MCI), brain atrophy is faster in women compared to men [10]. Females have been shown to have a higher prevalence and severity of neuropsychiatric symptoms associated with AD, and males have been shown to have more severe apathy [11].

Although there are several genome-wide association studies (GWAS) available which highlight genetic variants associated with AD, often the X chromosome is excluded from the analysis. The X chromosome is often excluded because of analytical challenges caused by unique features such as transcriptional silencing of one allele in females, hemizygosity in males and recombination patterns [12]. A PubMed search of chromosome X specific association analyses and AD from 2000 to present day identified only three relevant manuscripts. The first is a method to estimate risk on the X-chromosome but does not use X chromosome data directly [13]. The second investigated the number of single nucleotide variants on the X chromosome and compared this number between AD cases and controls, highlighting two genes *UBE2NL* and *ATXN3L* [14]. But only one study found have performed a chromosome X-wide association study (XWAS) for AD [15], but no associations reached genome-wide significance. A recent study investigating X chromosome gene expression found that several genes (including *GRIA3*, *GPRASP2*, and *GRIPAP1*) were associated with slower cognitive decline in women but not men. In contrast, X chromosome gene expression, like *UBL4A*, which encodes a protein folding factor and sorts proteins to the proteasome or to the endoplasmic reticulum, is associated with neuropathological tau burden in men but not women [16].

The aim of this study is to highlight single nucleotide polymorphisms (SNPs) and the most proximal genes to XWAS identified SNPs which are associated with AD risk on the X chromosome. To investigate potential sex differences in AD highlighted by genetics, we performed an XWAS (a genome-wide like association study focused on the X-chromosome) of AD risk by meta-analysing results from sex-stratified analyses. We conducted this in three collections of samples; (i) KRONOS/Tgen data which contains 994 AD cases and 572 controls, (ii) Brains for Dementia Research (BDR) data which contains 356 AD cases and 164 controls, and (iii) Religious Orders Study/Memory and Aging Project (ROSMAP) + Mount Sinai Brain Bank (MSBB) + Mayo Clinic Brain Bank (MAYO) data which contains 702 AD cases and 486 controls. The AD diagnosis in these data was pathologically confirmed. We used these studies to determine AD associated SNPs which replicate in at least two studies. We then meta-analysed the results with the view to increase power. In addition, the XWAS results for each study were used to perform a gene-based analysis to better identify associated genes with AD risk accounting for multiple, independent associations in the gene.

## Materials and methods

### Data description

The KRONOS/Tgen dataset is obtained from 21 National Alzheimer’s Coordinating Center (NACC) brain banks and from the Miami Brain Bank as previously described [17–20]. The criteria for inclusion were: self-defined ethnicity of European descent, neuropathologically confirmed AD or no neuropathology present, and age of death greater than 65. Neuropathological diagnosis was defined by board-certified neuropathologists according to the standard NACC protocols [21]. Samples derived from subjects with a clinical history of stroke, cerebrovascular disease, Lewy body dementia, or comorbidity with any other known neurological disease were excluded. AD or control neuropathology was confirmed by plaque and tangle assessment with 45% of the entire series undergoing Braak staging [22]. The cohort consists of 912 AD cases and 454 controls. Samples were de-identified and the study met human studies institutional review board and HIPPA regulations. This work is declared not human-subjects research and is IRB exempt under regulation 45 CFR 46. This data was imputed using the Michigan Imputation Server [23] using the TOPMed panel [24], and SNPs with an INFO score less than 0.7 were removed.

The Brains for Dementia Research (BDR, brainsfordementiaresearch.org.uk) data [25, 26] is a longitudinal cohort of dementia samples and controls. Currently there are approximately 1200 DNA samples from brain tissue or blood and this is expected to increase to 3200 samples with genetic information. BDR is a world-class brain tissue resource supported by the Alzheimer’s Society and Alzheimer’s Research UK establishing a network of brain banks in England and Wales. In addition, other data is collected related to cognition, general health, and lifestyle every 1–5 years. BDR data was imputed on the Michigan Imputation Server using Minimac4 pipeline and the TOPMed reference panel [24] is available through the Dementias Platform UK (DPUK, https://portal.dementiasplatform.uk). The genotyped cohort includes 354 cases confirmed with AD as the primary dementia (age at onset >65 years) and 163 cognitively normal controls without additional neuropathology; all diagnoses were neuropathologically confirmed [15].

We harmonised the Religious Orders Study/Memory and Aging Project [27] (ROSMAP), Mount Sinai Brain Bank (MSBB) and Mayo Clinic Brain Bank (MAYO) whole-genome sequenced data into one cohort which we then analysed together. Datasets were downloaded from the AMP-AD portal via the Synapse platform and https://www.radc.rush.edu. ROSMAP is a longitudinal study investigating AD and ageing [28, 29], MSBB has gene expression, genetic variant, neuropathological and proteomic data for brain specimens and MAYO is a cohort containing genetic, neuropathological, biochemistry and cell biology data. Quality control analysis was carried out in the combined data, as described in [30]. AD cases are defined using a subject’s clinical definition for AD and Braak score of 5 or 6 and controls are defined as those without a clinical AD diagnosis and Braak score less than or equal to 4. This sample contains 1188 individuals: 702 AD cases and 486 controls.

The demographic data for all cohorts is seen in Table 1. The age in cases and controls are comparable between KRONOS/Tgen, BDR and ROSMAP/MAYO/MSBB.

**Table 1:** Demographic summary for all cohorts.

Data	Demographics	Cases	Controls
KRONOS/Tgen	*N*	912	454
	Age	81.9 (8.75)	80.6 (8.80)
	Sex [M/F]	334/578	260/194
BDR	*N*	354	163
	Age	83.2 (8.50)	85.6 (9.68)
	Sex [M/F]	185/169	73/90
ROSMAP/MAYO/MSBB	*N*	702	486
	Age	86.4 (5.49)	84.8 (6.17)
	Sex [M/F]	223/479	182/304

### Quality control

In all data sets, the chromosome X data was QC’ed in males and females separately. The amount of missingness in individuals and SNPs were checked, but no missingness was found. SNPs out of Hardy-Weinberg Equilibrium (p < 1e-6), tested in females [31], were removed and SNPs with minor allele frequency (MAF) < 1% were also removed. In KRONOS/Tgen 225,873 and 227,323 SNPs, in BDR 228,716 and 230,052 SNPs and in ROSMAP/MAYO/MSBB 167,018 and 169,794 SNPs were retained, in males and females respectively. SNPs in all datasets are genome build 38.

### Chromosome X-wide Association Studies (XWAS)

The XWAS was carried out in males and females separately in Plink v1.9 [32, 33] using option *--xchr-model 2*. The models were adjusted for age and principal components (PCs); 5 PCs were used for KRONOS/Tgen and 10 PCs were used in BDR and ROSMAP/MAYO/MSBB. The number of PCs necessary for adjustment was determined from visual inspection of PC plots.

The XWAS from males and females were meta-analysed together using GWAMA [34] which also reports differentiation and heterogeneity between results in males and females. These results were represented using a Manhattan plot using the manhattan() function in R [35]. An FDR multiple testing correction was applied to identify significantly associated SNPs.

### Meta-analysis of AD XWAS

The XWAS were meta-analysed together using METAL [36], results in males and females were meta-analysed separately and then GWAMA was used to join results in males and females. These results were represented using a Manhattan plot using the manhattan() function in R [35]. An FDR multiple testing correction was applied to identify significantly associated SNPs.

### Gene-based analysis

A gene-based analysis of the XWAS was carried out in MAGMA v.1.08 [37], SNPs were assigned to genes based on gene locations from the NCBI site using a window of 35 kb upstream and 10 kb downstream and the original data was used to estimate linkage disequilibrium (LD) between SNPs. The mean-chi2 approach was used, which averages the effect of SNPs in the gene. The KRONOS/Tgen and BDR data annotates to 800 genes and ROSMAP/MAYO/MSBB annotates to 665 genes. An FDR multiple testing correction was applied to identify significantly associated genes.

### Using expression data to gain insights into genes of interest

To gain insights into how these chromosome X putative risk genes may contribute to AD, we searched a series of publicly open datasets, including our own, containing expression data for these genes from bulk and single-cell RNA-seq datasets from human and mouse [20, 38–49]. We have also examined RNA samples of AD mouse models [50–52]. STRING database (string-db.org) was used to assess Protein-Protein Interaction Networks for the identified putative genes. Ingenuity analysis (digitalinsights.qiagen.com) was performed with candidate genes across all mammalian species for tissues and cell types curated in Ingenuity.

## Results

### XWAS results

The XWAS results in the KRONOS/Tgen data, with males and females meta-analysed together are presented in Supplementary Fig. 1. There are no SNPs with association above the chromosome-wide significant threshold, but there are several peaks which reach suggestive significance (p < 1.1e-03). The GWAMA software provides a *p* value for a sex differentiated effect. Of the top SNPs presented in Supplementary Table 1, rs5910591 has a differential effect between males and females (*p* = 3.2e-05), the effect is driven by females (OR = 0.48, *p* = 2.4e-05, Ref/Alt Allele=G/A, MAF = 0.20), and there is no significant effect in males (0.82, *p* = 0.10, Ref/Alt Allele= G/A, MAF = 0.21).

The Manhattan plot of the BDR data with males and females combined is seen in Supplementary Fig. 2. Similarly, to the KRONOS/Tgen data, no SNPs reach chromosome-wide significance but a number reach suggestive significance (1.1e-03). The top SNPs from these peaks can be seen in Supplementary Table 2. The top SNPs from BDR are different to those in KRONOS/Tgen but rs186553004 and rs5913102 both replicate (OR = 0.39, *p* = 0.031, Ref/Alt Allele=G/C, MAF = 0.02; OR = 0.69, *p* = 0.009, Ref/Alt Allele=C/T, MAF = 0.18 respectively).

The ROSMAP/MAYO/MSBB XWAS results in males and females combined is seen in Supplementary Fig. 3. There are several suggestive SNPs (1.4e-03) but none are chromosome-wide significant, the top SNPs from these peaks are seen in Supplementary Table 3. The ROSMAP/MAYO/MSBB XWAS identifies another set of SNPs associated with AD, however, the *SH3BGRL* gene is identified by both ROSMAP/MAYO/MSBB and KRONOS/Tgen and *IL1RAPL1* is highlighted by both ROSMAP/MAYO/MSBB and BDR. In addition to these replicated genes, SNPs rs5913102 and rs12006935 identified previously are replicated in ROSMAP (OR = 0.78, *p* = 0.008, Ref/Alt Allele=C/T, MAF = 0.19; OR = 0.79, *p* = 0.028, Ref/Alt Allele=A/C, MAF = 0.23 respectively).

### Meta-analysis

**Fig. 1:**
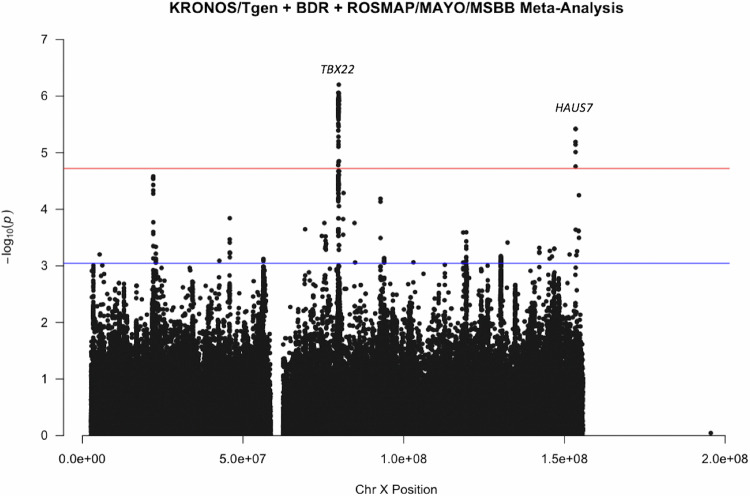
Manhattan Plot of KRONOS/Tgen + BDR + ROSMAP/MAYO/MSBB XWAS. Each dot represents a SNP, the *x*-axis is the SNPs base position and the *y*-axis is the *p*-value (–log10(*p*)), the red line shows the chromosome X wide significance threshold and the blue line shows the suggestive significance threshold.

Since there was apparent overlap between results, we meta-analysed all three cohorts together. The meta-analysis produced results for 264,793 SNPs. The results can be seen in Fig. 1, two peaks can be observed which are chromosome-wide significant based on an FDR correction of chromosome X SNPs. Table 2 shows all SNPs which were highlighted by a single cohort which also replicate in the meta-analysis; the effect sizes and *p* values in each single cohort and also from the meta-analysis are presented. The Manhattan plots in males and females separately are seen in Supplementary Figs. 4 and 5 respectively. There is a peak in females which reaches chromosome-wide significance based on an FDR correction which is not seen in males, these SNPs map to gene *NXF5*.

**Table 2:** SNPs from single studies which replicate in meta-analysis XWAS (KRONOS/Tgen + BDR + ROSMAP/MAYO/MSBB).

SNP	BP	BDR	KRONOS/Tgen	ROSMAP/MAYO/MSBB	Meta-analysis	Nearest Gene
		OR (P)				
rs4827693	146138708	1.06 (0.621)	**1.33** **(5.8e-05)**	0.94 (0.400)	**1.12** **(0.018)**	*TMEM257*
rs12848641	101367905	1.13 (0.511)	**0.69** **(1.0e-04)**		**0.76** **(0.002)**	*BTK*
rs186553004	81250064	**0.39** **(0.031)**	**0.35** **(0.0005)**		**0.36** **(5.2e-05)**	*SH3BGRL*
rs113157993	82089923	0.94 (0.803)	0.96 (0.78)	**0.52** **(0.0003)**	**0.79** **(0.03)**	*SH3BGRL*
rs5913102	79809325	**0.69** **(0.009)**	**0.74** **(0.0003)**	**0.78** **(0.008)**	**0.75** **(6.3e-07)**	*TBX22*
rs2089596385	153481028	**1.86** **(3.6e-06)**			**1.85** **(3.8e-06)**	*HAUS7*
rs12006935	22857207	**0.52** **(6.9e-05)**	0.91 (0.332)	**0.79** **(0.028)**	**0.79** **(0.0005)**	*DDX53*
rs9969903	93503072	1.04 (0.707)	0.94 (0.394)	**0.73** **(4.4e-05)**	**0.88** **(0.0057)**	*NAP1L3*
rs112073726	75303891			**0.55** **(0.0001)**	**0.54** **(0.0002)**	*UPRT*
rs147450445	145399999	0.98 (0.954)	0.79 (0.182)	**0.51** **(0.0003)**	**0.68** **(0.002)**	*SPANXN1*
rs137983810	29572683		1.05 (0.765)	**0.52** **(0.0003)**	**0.76** **(0.038)**	*IL1RAPL1*
rs6628450	29551448	**0.36** **(4.2e-05)**	0.77 (0.157)	1.21 (0.234)	0.81 (0.06)	*IL1RAPL1*

Numbers in bold are effects with a *p* value less than 0.05.

**Fig. 2:**
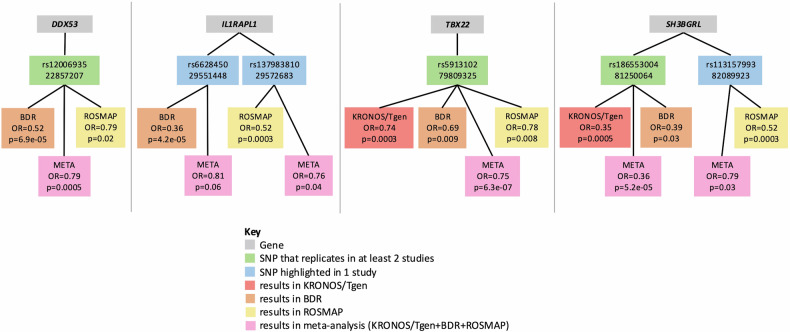
Summary of findings across all cohorts. The four panels represent each of the four genes (grey) highlighted in this study, the relevant identified SNPs (blue/green) for each genes, and the effect sizes and *p*-values in the different cohorts (red/orange/yellow) and the meta-analysis of all cohorts (pink).

The results across all cohorts are summarised in Fig. 2, this highlights that evidence is strongest for 4 genes; *DDX53*, *IL1RAPL1*, *TBX22* and *SH3BGRL*, which show replication in at least two independent cohorts. So, although individual SNP association *p* values are only suggestive, we can be more confident in these findings as they replicate across multiple studies. In addition, in the meta-analysed data SNP rs5913102 in *TBX22* reaches chromosome-wide significance based on an FDR correction. The LocusZoom [53] plots for these four genes in the meta-analysed (KRONOS/Tgen + BDR + ROSMAP/MAYO/MSBB) data is seen in Supplementary Fig. 6.

SNPs in Table 2 were uploaded to RegulomeDB [54], SNPs rs5913102 and rs12848641 had rank “1f” indicating that these SNPs are likely to be located in a functional region and affect transcription factor binding. All other SNPs had rank > 4 suggesting minimal binding evidence. We also computed a combined annotation dependent depletion (CADD) score [55] for each of these variants which integrates several diverse annotations, but no SNPs had a score > 20 suggesting that variants may not be functional.

We have also found SNPs significantly associated with AD in the genes which have been identified in [16], which also used ROSMAP but gene expression data. We replicated signals in three genes in the meta-analysis of all three cohorts, namely *GRIA3* (rs6649016, OR = 1.15, *p* = 0.0028, Ref/Alt Allele=A/G), *GRIPAP1* (rs5906732, OR = 1.53, *p* = 0.028, Ref/Alt Allele=T/C) and *UBL4A* (rs45463798, OR = 1.16, *p* = 0.05, Ref/Alt Allele=A/G).

The SNPs presented in Table 2 have effect sizes in the same direction in males and females, see Supplementary Table 4. For the SNPs replicating across multiple studies we also investigated the impact of *APOE* status on these associations, by adjusting for both number of APOEe4 alleles and the interaction between the SNP and number of *APOE* e4 alleles, the *p* value for this model is presented in Supplementary Table 4. In general, the *p*-value adjusted for *APOE* status has only changed slightly, with the largest change for the *SH3BGRL* gene (*p* = 5.2e-5, p_adj_ = 0.014). We have identified SNPs in *NXF5* gene to be chromosome-wide significant in females but not in males (rs5944989 MAF = 0.60 and 0.58, OR = 0.62 and 0.98, *p* = 1.1e-05 and 0.830, in females and males, respectively, Ref/Alt Allele=A/G), see Table 3.

**Table 3:** Top heterogenous SNPs between males and females in the Meta-Analysis XWAS (KRONOS/Tgen+BDR + ROSMAP/MAYO/MSBB).

SNP	BP	Males		Females		Sex heterogeneity P	Nearest Gene
		OR	P	OR	P		
rs111481225	62858686	0.51	0.004	2.18	0.002	2.4e-05	*SPIN4*
rs111938044	64282895	0.36	0.002	2.37	0.003	2.4e-05	*LOC105373237*
rs112957841	65258199	0.29	0.002	2.13	0.007	3.8e-05	*ZC3H12B*
rs144256274	147134444	1.34	0.059	0.52	0.0004	8.7e-05	*LOC105373347*
rs5944989	101807831	0.98	0.830	0.62	1.1e-05	0.0005	*NXF5*

When meta-analysing males and females using GWAMA, a sex heterogeneity *p*-value is computed; the top SNPs with the smallest *p*-values for heterogeneity are also seen in Table 3. These SNPs show an opposite effect direction in males and females.

### Gene-based analysis

The gene-based analysis in the KRONOS/Tgen and ROSMAP/MAYO/MSBB data does not provide any genes which surpass the gene-wide threshold (based upon the total number of chromosome X genes analysed). In the KRONOS/Tgen data, the third most significant gene is *SLC25A5* (*p* = 0.0048, p_fdr_ = 0.74) which was also highlighted as a proximal gene to a SNP identified in the XWAS. In the BDR data, there were two genes which reached gene-wide significance, these were *BGN* and *HAUS7* (p_fdr _= 0.004 for both genes). *HAUS7* was also identified as chromosome-wide significant from the XWAS in the meta-analysed data. A gene-based analysis from the meta-analysis also did not produce any gene-wide significant results.

### Using expression data to gain insights into genes of interest

**Fig. 3:**
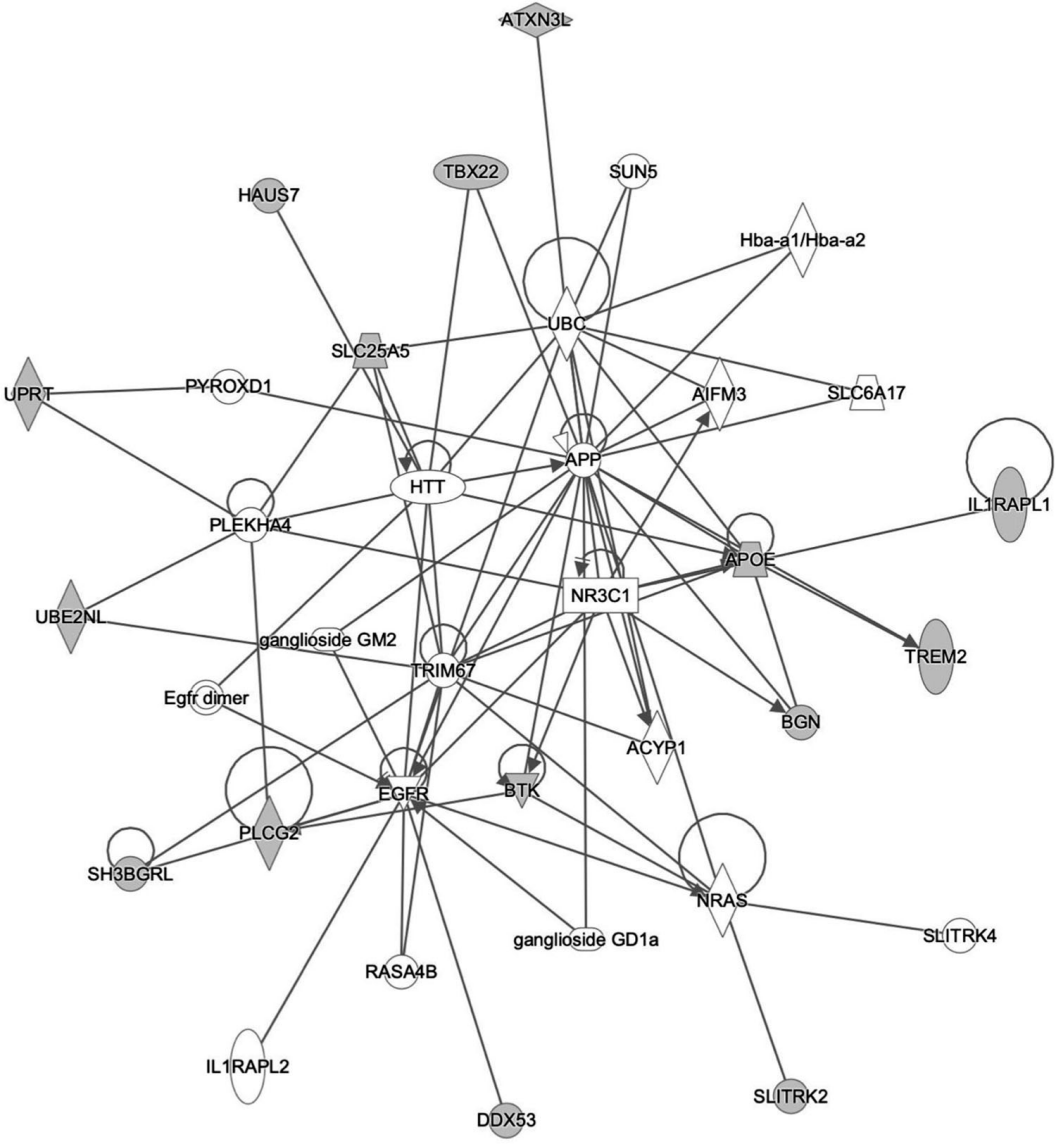
The Ingenuity Pathway Analysis (IPA) explores candidate genes across various mammalian species within tissues and cell types catalogued in Ingenuity. Input genes are denoted by gene symbols encircled with grey filled nodes, while solid lines signify direct interactions, such as protein-protein interactions or phosphorylation events.

We investigated the meta-analysis XWAS significant genes (Table 2) for their relevance to neurodegeneration by searching several public datasets containing expression data from bulk and single-cell RNA-seq datasets from human and mouse samples to assess conservation of gene responses between species. We saw *DDX53* was expressed at low levels in several cell types across the human brain but showed highest expression in astrocytes (Supplementary Fig. 7A). In some human AD studies *DDX53* showed increased expression in AD compared to age-matched controls (Supplementary Fig. 7B). *IL1RAPL1* was expressed at highest levels in human oligodendrocytes and neurons (Supplementary Fig. 8A) and showed decreased expression in mouse models of AD compared to age-matched wild-type mice (Supplementary Fig. 8C). *SH3BGRL* was expressed at the highest levels in human microglia (Supplementary Fig. 9A), and showed decreased expression in AD compared to age-matched controls in one data set (Supplementary Fig. 9B), and increased expression in another (Supplementary Fig. 9C). In mouse models of AD, *SH3BGRL* showed increased expression compared to age-matched controls (Supplementary Fig. 9D, E). Data was not available for *TBX22*, and so was likely lowly expressed in the datasets examined. We saw that these genes of interest could be linked to known familial neurodegenerative disease genes (*APP* and *HTT*), and AD risk genes (*APOE* and *PLCG2*) from prior experimental studies using the Ingenuity database (Fig. 3).

*BTK* is an additional gene which was highlighted in only one study but show interesting expression results. *BTK* was expressed at the highest levels in human microglia (Supplementary Fig. 10A). *BTK* showed increased expression in AD compared to age-matched controls (Supplementary Fig. 10B), and increased expression in mouse models of AD (Supplementary Fig. 10E). In the STRING database, the *BTK-based* network includes *PLCG2, SYK, TLR4, TGFB1* and *TREM2* genes which belong to microglial pathways important in AD (Supplementary Fig. 11). Thus, the new chromosome X genes we have identified are likely to contribute to AD by modifying existing pathways that are known to control AD risk.

## Discussion

This study, which uses a pathologically confirmed diagnosis of AD, identifies four potential genes, *DDX53*, *IL1RAPL1*, *TBX22* and *SH3BGRL*, associated with AD, which replicate across at least two of the sub-studies.

*TBX22* has previously been shown to be associated with cleft lip and cleft palate [56]. The SNP in *TBX22* has a RegulomeDB [54] rank of “1f” indicating the SNPs likelihood of being in a functional region and affects transcription factor binding.

*SH3BGRL* has been linked to Parkinson’s disease, where higher expression is shown in cases compared to controls [57] and is highly expressed in breast cancers [58]. In addition, a proteome analysis in AD has identified increased SH3 protein in the brain [59].

*DDX53* is an intronless gene which is linked to Autism Spectrum Disorder (ASD), although the *DDX53* mutations were shown to have no effect on synaptic transmission [60].

*IL1RAPL1* is a synaptic adhesion molecule located at the postsynaptic membrane, it regulates dendrite formation and impacts activity of IL-1β on dendrite morphology [61]. Literature suggests that there are other genes in the IL1 family that have some relevance to neurodegenerative or brain disorders. *IL1RAP* is highly expressed in the brain [62] and SNPs located in *IL1RAP* were found to be associated with longitudinal change in brain amyloid [63]. There were also associations found between the most significant SNP in *IL1RAP* and progression from MCI to AD, cognitive decline, temporal cortex atrophy and microglial activity [63]. rs1921622, a variant in *IL1RL1* has been shown to lower the risk effects of *APOE*-ε4 in female AD patients by lowering soluble ST2 [64].

Several genes were identified as being associated with sex-differentiated effects. *NXF5* was found to be chromosome-wide significant in females but not in males. *NXF5* has been linked to intellectual disability [65] and is known to be involved in brain development [66]. SNPs in *SPIN4, LOC105373237, ZC3H12B* and *LOC105373347* genes were identified as the most significant sex heterogeneous SNPs in the XWAS meta-analysis. The *SPIN4* gene inhibits cell proliferation, binds specific histone modifications and negatively regulates body growth [67]. *ZC3H12B* has been identified as being associated with AD using a Bayesian genome-wide transcriptome-wide association study [68].

Three genes identified in a previous study [16] were replicated from the meta-analysis XWAS of all three cohorts; these genes were *GRIA3*, *GRIPAP1* and *UBL4A*. As well as being associated with slower cognitive decline in women but not men, *GRIA3* is known to be involved in memory and learning and is highly correlated to *HLA-DRB5*, which is associated with AD [69].

Two additional gene-wide significant genes were identified in the BDR data in the gene-based analysis; these genes are *BGN* and *HAUS7. BGN* has been associated with amyloid metabolism in AD [70], inflammatory state in obesity and type 2 diabetes [71] and is known to be a central gene in a network in the brain in response to fructose consumption [72]. *HAUS7* is necessary for cytokinesis and regulates mitotic spindle and centrosome integrity (https://www.ncbi.nlm.nih.gov/). SNPs in *HAUS7* also reach chromosome-wide significance after FDR multiple testing correction in the meta-analysis of all three cohorts.

*BTK* was found to be strongly connected to *PLCG2* [73]. *PLCG2* activation leads to the B cell receptor (BCR) signalling and *BTK* is in the BCR signalling complex. In the protein network, Toll Like Receptor 4 (TLR4) connects *BGN* to the *BTK* network [73, 74] including the *PLCG2* pathway and epigenetic silencing of the immunosuppressive response. *PLCG2* is a well validated AD risk gene [75, 76]. The *BTK-BGN* network includes strong microglial genes (*PLCG2, SYK, TLR4, TGFB1*) and *TREM2* links strongly to these [76].

*DDX53* shows highest expression in astrocytes, *IL1RAPL1* is most highly expressed in oligodendrocytes and neurons and *SH3BGRL* is most highly expressed in microglia. Collectively, the expression data suggests that the putative chromosome X risk genes act through different cell types and pathways to modulate risk for AD, with some genes increasing risk and some being protective.

The strength of this study is the utilisation of available chromosome X data in the KRONOS/Tgen, BDR and ROSMAP/MAYO/MSBB data in relation to AD, which until now has been understudied. It also uses three independent cohorts with a pathologically confirmed phenotype to investigate potential replication and increase power by meta-analysing independent XWAS together.

The limitation of this study is the relatively small sample size of the available studies; however, we have attempted to improve power by meta-analysing these cohorts together. Despite the small sample sizes, we report consistent results across studies which reinforce our findings.

In conclusion, this study has highlighted several potential target genes on chromosome X associated with AD risk which may be relevant for further study, with the end goal of identifying differences in AD progression between males and females and potentially developing sex-stratified therapeutics.

## Supplementary information

Supplementary Material

## Data Availability

The XWAS summary statistics for the meta-analysis of KRONOS/Tgen and BDR is available at the DRI GitHub repository (https://github.com/UKDRI/XWAS_AD_summary_stats). The Manhattan plot for this meta-analysis is presented in Supplementary Fig. 12. Mouseac, this paper and Matarin et al. [51]: www.mouseac.org WGCNA (Langfelder and Horvath, 2008): https://horvath.genetics.ucla.edu/html/CoexpressionNetwork/Rpackages/WGCNA/index.html (accessed September 2018) Braineac (Ramasamy et al., 2014): www.braineac.org (accessed September 2018) 1,000 genomes (Genomes Project et al., 2015): www.1000genomes.org and http://www.internationalgenome.org (accessed September 2018) MAGMA de Leeuw et al. [37]: www.ctg.cncr.nl/software/magma (accessed May 2019) Coloc, version 3.1, (Giambartolomei et al., 2014): https://github.com/chr1swallace/coloc (accessed September 2018) ROS/MAP (Bennett et al. [29]; Bennett et al. [29]; De Jager et al., 2018): https://www.synapse.org/#!Synapse:syn3219045 (accessed September 2018) i-CisTarget (Imrichova et al., 2015): https://gbiomed.kuleuven.be/apps/lcb/i-cisTarget (accessed September 2018) GTEx V6 gene expression (Consortium GT, 2015): https://gtexportal.org/home (accessed September 2018) Coexp (Botia et al., 2017): https://github.com/juanbot/CoExpNets (accessed September 2018) Myeloid landscape datasets (Friedman et al., 2018): http://research-pub.gene.com/BrainMyeloidLandscape/# (accessed June 2019).

## References

[1] Alzheimer’s Association. 2023 Alzheimer’s disease facts and figures. Alzheimer’s Dement. 2023. https://www.alz.org/alzheimers-dementia/factsfigures#:~:text=The%20lifetime%20risk%20for%20Alzheimer's,valued%20at%20nearly%20%24350%20billion.

[2] Hebert LE, Weuve J, Scherr PA, Evans DA. Alzheimer disease in the United States (2010-2050) estimated using the 2010 census. Neurology. 2013;80:1778–83. 23390181 10.1212/WNL.0b013e31828726f5 PMC3719424

[3] Mielke MM. Sex and gender differences in Alzheimer’s disease dementia. Psychiatr Times. 2018;35:14–7. 30820070 PMC6390276

[4] Plassman BL, Langa KM, Fisher GG, Heeringa SG, Weir DR, Ofstedal MB, et al. Prevalence of dementia in the United States: the aging, demographics, and memory study. Neuroepidemiology. 2007;29:125–32. 17975326 10.1159/000109998 PMC2705925

[5] Roberts RO, Knopman DS, Mielke MM, Cha RH, Pankratz VS, Christianson TJ, et al. Higher risk of progression to dementia in mild cognitive impairment cases who revert to normal. Neurology. 2014;82:317–25. 24353333 10.1212/WNL.0000000000000055 PMC3929198

[6] Guo L, Zhong MB, Zhang L, Zhang B, Cai D. Sex differences in Alzheimer’s disease: insights from the multiomics landscape. Biol Psychiatry. 2022;91:61–71. 33896621 10.1016/j.biopsych.2021.02.968 PMC8996342

[7] Gilsanz P, Lee C, Corrada MM, Kawas CH, Quesenberry CP, Whitmer RA. Reproductive period and risk of dementia in a diverse cohort of health care members. Neurology. 2019;92:e2005–e14. 30923235 10.1212/WNL.0000000000007326 PMC6511081

[8] Gong J, Harris K, Peters SAE, Woodward M. Reproductive factors and the risk of incident dementia: a cohort study of UK Biobank participants. PLoS Med. 2022;19:e1003955. 35381014 10.1371/journal.pmed.1003955 PMC8982865

[9] Farrer LA, Cupples LA, Haines JL, Hyman B, Kukull WA, Mayeux R, et al. Effects of age, sex, and ethnicity on the association between apolipoprotein E genotype and Alzheimer disease: a meta-analysis. JAMA. 1997;278:1349–56. 9343467 10.1001/jama.1997.03550160069041

[10] Ferretti MT, Iulita MF, Cavedo E, Chiesa PA, Schumacher Dimech A, Santuccione Chadha A, et al. Sex differences in Alzheimer disease - the gateway to precision medicine. Nat Rev Neurol. 2018;14:457–69. 29985474 10.1038/s41582-018-0032-9

[11] Eikelboom WS, Pan M, Ossenkoppele R, Coesmans M, Gatchel JR, Ismail Z, et al. Sex differences in neuropsychiatric symptoms in Alzheimer’s disease dementia: a meta-analysis. Alzheimers Res Ther. 2022;14:48. 35379344 10.1186/s13195-022-00991-z PMC8978393

[12] Gorlov IP, Amos CI. Why does the X chromosome lag behind autosomes in GWAS findings? PLoS Genet. 2023;19:e1010472. 36848382 10.1371/journal.pgen.1010472 PMC9997976

[13] Armon C, Wolfson S, Margalit R, Avraham L, Bugen Y, Cohen A, et al. Estimating the X chromosome-mediated risk for developing Alzheimer’s disease. J Neurol. 2022;269:2479–85. 34609600 10.1007/s00415-021-10826-w

[14] Gomez-Ramos A, Podlesniy P, Soriano E, Avila J. Distinct X-chromosome SNVs from some sporadic AD samples. Sci Rep. 2015;5:18012. 26648445 10.1038/srep18012 PMC4673451

[15] Young J, Gallagher E, Koska K, Guetta-Baranes T, Morgan K, Thomas A, et al. Genome-wide association findings from the brains for dementia research cohort. Neurobiol Aging. 2021;107:159–67. 34183186 10.1016/j.neurobiolaging.2021.05.014

[16] Davis EJ, Solsberg CW, White CC, Miñones-Moyano E, Sirota M, Chibnik L, et al. Sex-specific association of the X chromosome with cognitive change and tau pathology in aging and Alzheimer Disease. JAMA Neurol. 2021;78:1249–54. 34424272 10.1001/jamaneurol.2021.2806 PMC8383157

[17] Corneveaux JJ, Myers AJ, Allen AN, Pruzin JJ, Ramirez M, Engel A, et al. Association of CR1, CLU and PICALM with Alzheimer’s disease in a cohort of clinically characterized and neuropathologically verified individuals. Hum. Mol. Genet. 2010;19:3295–301. 20534741 10.1093/hmg/ddq221 PMC2908469

[18] Myers AJ, Gibbs JR, Webster JA, Rohrer K, Zhao A, Marlowe L, et al. A survey of genetic human cortical gene expression. Nat. Genet. 2007;39:1494–9. 17982457 10.1038/ng.2007.16

[19] Petyuk, Chang VA, Ramirez-Restrepo R, Beckmann M, Henrion MYR ND, Piehowski PD, et al. The human brainome: network analysis identifies HSPA2 as a novel Alzheimer disease target. Brain. 2018;141:2721–39. 30137212 10.1093/brain/awy215 PMC6136080

[20] Webster JA, Gibbs JR, Clarke J, Ray M, Zhang W, Holmans P, et al. Genetic control of human brain transcript expression in Alzheimer disease. Am J Hum Genet. 2009;84:445–58. 19361613 10.1016/j.ajhg.2009.03.011 PMC2667989

[21] Beekly DL, Ramos EM, van Belle G, Deitrich W, Clark AD, Jacka ME, et al. The National Alzheimer’s Coordinating Center (NACC) Database: an Alzheimer disease database. Alzheimer Dis Assoc Disord. 2004;18:270–7. 15592144

[22] Braak H, Braak E. Staging of alzheimer’s disease-related neurofibrillary changes. Neurobiol Aging. 1995;16:271–8. 7566337 10.1016/0197-4580(95)00021-6

[23] Das S, Forer L, Schonherr S, Sidore C, Locke AE, Kwong A, et al. Next-generation genotype imputation service and methods. Nat Genet. 2016;48:1284–7. 27571263 10.1038/ng.3656 PMC5157836

[24] Taliun D, Harris DN, Kessler MD, Carlson J, Szpiech ZA, Torres R, et al. Sequencing of 53,831 diverse genomes from the NHLBI TOPMed Program. Nature. 2021;590:290–9. 33568819 10.1038/s41586-021-03205-y PMC7875770

[25] Francis PT, Costello H, Hayes GM. Brains for dementia research: evolution in a longitudinal brain donation cohort to maximize current and future value. J Alzheimers Dis. 2018;66:1635–44. 30452415 10.3233/JAD-180699 PMC6294579

[26] Francis PT, Hayes GM, Costello H, Whitfield DR. Brains for dementia research: the importance of cohorts in brain banking. Neurosci Bull. 2019;35:289–94. 30604278 10.1007/s12264-018-0327-2 PMC6426925

[27] Bennett DA, Buchman AS, Boyle PA, Barnes LL, Wilson RS, Schneider JA. Religious orders study and rush memory and aging project. J Alzheimer’s Dis. 2018;64:S161–S89. 29865057 10.3233/JAD-179939 PMC6380522

[28] Bennett D, Schneider J, Arvanitakis Z, Wilson R. Overview and findings from the religious orders study. Curr Alzheimer Res. 2011;9:628–45. 10.2174/156720512801322573 PMC3409291 22471860

[29] Bennett DA, Schneider JA, Buchman AS, Barnes LL, Boyle PA, Wilson RS. Overview and findings from the rush memory and aging project. Curr Alzheimer Res. 2012;9:646–63. 22471867 10.2174/156720512801322663 PMC3439198

[30] Leonenko G, Baker E, Stevenson-Hoare J, Sierksma A, Fiers M, Williams J, et al. Identifying individuals with high risk of Alzheimer’s disease using polygenic risk scores. Nat Commun. 2021;12:4506. 34301930 10.1038/s41467-021-24082-z PMC8302739

[31] Konig IR, Loley C, Erdmann J, Ziegler A. How to include chromosome X in your genome-wide association study. Genet Epidemiol. 2014;38:97–103. 24408308 10.1002/gepi.21782

[32] Purcell S, Neale B, Todd-Brown K, Thomas L, Ferreira MA, Bender D, et al. PLINK: a tool set for whole-genome association and population-based linkage analyses. Am J Hum Genet. 2007;81:559–75. 17701901 10.1086/519795 PMC1950838

[33] Chang CC, Chow CC, Tellier LC, Vattikuti S, Purcell SM, Lee JJ. Second-generation PLINK: rising to the challenge of larger and richer datasets. Gigascience. 2015;4:7. 25722852 10.1186/s13742-015-0047-8 PMC4342193

[34] Mägi R, Morris AP. GWAMA: software for genome-wide association meta-analysis. BMC Bioinform. 2010;11:288. 10.1186/1471-2105-11-288 PMC2893603 20509871

[35] R Development Core Team. R: A language and environment for statistical computing. R Foundation for Statistical Computing. 2008.

[36] Willer CJ, Li Y, Abecasis GR. METAL: fast and efficient meta-analysis of genomewide association scans. Bioinformatics. 2010;26:2190–1. 20616382 10.1093/bioinformatics/btq340 PMC2922887

[37] de Leeuw CA, Mooij JM, Heskes T, Posthuma D. MAGMA: generalized gene-set analysis of GWAS data. PLoS Comput Biol. 2015;11:e1004219. 25885710 10.1371/journal.pcbi.1004219 PMC4401657

[38] Zhang B, Gaiteri C, Bodea LG, Wang Z, McElwee J, Podtelezhnikov AA, et al. Integrated systems approach identifies genetic nodes and networks in late-onset Alzheimer’s disease. Cell. 2013;153:707–20. 23622250 10.1016/j.cell.2013.03.030 PMC3677161

[39] Berchtold NC, Cribbs DH, Coleman PD, Rogers J, Head E, Kim R, et al. Gene expression changes in the course of normal brain aging are sexually dimorphic. Proc Natl Acad Sci USA. 2008;105:15605–10. 18832152 10.1073/pnas.0806883105 PMC2563070

[40] Srinivasan K, Friedman BA, Etxeberria A, Huntley MA, van der Brug MP, Foreman O, et al. Alzheimer’s patient microglia exhibit enhanced aging and unique transcriptional activation. Cell Rep. 2020;31:107843. 32610143 10.1016/j.celrep.2020.107843 PMC7422733

[41] Olah M, Patrick E, Villani AC, Xu J, White CC, Ryan KJ, et al. A transcriptomic atlas of aged human microglia. Nat Commun. 2018;9:539. 29416036 10.1038/s41467-018-02926-5 PMC5803269

[42] Zhang Y, Chen K, Sloan SA, Bennett ML, Scholze AR, O’Keeffe S, et al. An RNA-sequencing transcriptome and splicing database of glia, neurons, and vascular cells of the cerebral cortex. J Neurosci. 2014;34:11929–47. 25186741 10.1523/JNEUROSCI.1860-14.2014 PMC4152602

[43] Zhang Y, Sloan SA, Clarke LE, Caneda C, Plaza CA, Blumenthal PD, et al. Purification and characterization of progenitor and mature human astrocytes reveals transcriptional and functional differences with mouse. Neuron. 2016;89:37–53. 26687838 10.1016/j.neuron.2015.11.013 PMC4707064

[44] Morabito S, Miyoshi E, Michael N, Shahin S, Martini AC, Head E, et al. Single-nucleus chromatin accessibility and transcriptomic characterization of Alzheimer’s disease. Nat Genet. 2021;53:1143–55. 34239132 10.1038/s41588-021-00894-z PMC8766217

[45] Sala Frigerio C, Wolfs L, Fattorelli N, Thrupp N, Voytyuk I, Schmidt I, et al. The major risk factors for Alzheimer’s disease: age, sex, and genes modulate the microglia response to Abeta plaques. Cell Rep. 2019;27:1293–306.e6. 31018141 10.1016/j.celrep.2019.03.099 PMC7340153

[46] Lake BB, Chen S, Sos BC, Fan J, Kaeser GE, Yung YC, et al. Integrative single-cell analysis of transcriptional and epigenetic states in the human adult brain. Nat Biotechnol. 2018;36:70–80. 29227469 10.1038/nbt.4038 PMC5951394

[47] Swarup V, Chang TS, Duong DM, Dammer EB, Dai J, Lah JJ, et al. Identification of conserved proteomic networks in neurodegenerative dementia. Cell Rep. 2020;31:107807. 32579933 10.1016/j.celrep.2020.107807 PMC8221021

[48] Wang Y, Cella M, Mallinson K, Ulrich JD, Young KL, Robinette ML, et al. TREM2 lipid sensing sustains the microglial response in an Alzheimer’s disease model. Cell. 2015;160:1061–71. 25728668 10.1016/j.cell.2015.01.049 PMC4477963

[49] Ulland TK, Song WM, Huang SC, Ulrich JD, Sergushichev A, Beatty WL, et al. TREM2 maintains microglial metabolic fitness in Alzheimer’s disease. Cell. 2017;170:649–63.e13. 28802038 10.1016/j.cell.2017.07.023 PMC5573224

[50] Orre M, Kamphuis W, Osborn LM, Jansen AHP, Kooijman L, Bossers K, et al. Isolation of glia from Alzheimer’s mice reveals inflammation and dysfunction. Neurobiol Aging. 2014;35:2746–60. 25002035 10.1016/j.neurobiolaging.2014.06.004

[51] Matarin M, Salih DA, Yasvoina M, Cummings DM, Guelfi S, Liu W, et al. A genome-wide gene-expression analysis and database in transgenic mice during development of amyloid or tau pathology. Cell Rep. 2015;10:633–44. 25620700 10.1016/j.celrep.2014.12.041

[52] Salih DA, Bayram S, Guelfi S, Reynolds RH, Shoai M, Ryten M, et al. Genetic variability in response to amyloid beta deposition influences Alzheimer’s disease risk. Brain Commun. 2019;1:fcz022. 32274467 10.1093/braincomms/fcz022 PMC7145452

[53] Boughton AP, Welch RP, Flickinger M, VandeHaar P, Taliun D, Abecasis GR, et al. LocusZoom.js: interactive and embeddable visualization of genetic association study results. Bioinformatics. 2021;37:3017–8. 33734315 10.1093/bioinformatics/btab186 PMC8479674

[54] Boyle AP, Hong EL, Hariharan M, Cheng Y, Schaub MA, Kasowski M, et al. Annotation of functional variation in personal genomes using RegulomeDB. Genome Res. 2012;22:1790–7. 22955989 10.1101/gr.137323.112 PMC3431494

[55] Kircher M, Witten DM, Jain P, O’Roak BJ, Cooper GM, Shendure J. A general framework for estimating the relative pathogenicity of human genetic variants. Nat Genet. 2014;46:310–5. 24487276 10.1038/ng.2892 PMC3992975

[56] Marcano AC, Doudney K, Braybrook C, Squires R, Patton MA, Lees MM, et al. TBX22 mutations are a frequent cause of cleft palate. J Med Genet. 2004;41:68–74. 14729838 10.1136/jmg.2003.010868 PMC1757272

[57] Werner CJ, Heyny-von Haussen R, Mall G, Wolf S. Proteome analysis of human substantia nigra in Parkinson’s disease. Proteome Sci. 2008;6:8. 18275612 10.1186/1477-5956-6-8 PMC2265686

[58] Li H, Zhang M, Wei Y, Haider F, Lin Y, Guan W, et al. SH3BGRL confers innate drug resistance in breast cancer by stabilizing HER2 activation on cell membrane. J Exp Clin Cancer Res. 2020;39:81. 32381043 10.1186/s13046-020-01577-z PMC7204297

[59] Shiozaki A, Tsuji T, Kohno R, Kawamata J, Uemura K, Teraoka H, et al. Proteome analysis of brain proteins in Alzheimer’s disease: subproteomics following sequentially extracted protein preparation. J Alzheimer’s Dis. 2004;6:257–68. 15201480 10.3233/JAD-2004-6306

[60] Faheem M, Deneault E, Alexandrova R, Rodrigues DC, Pellecchia G, Shum C, et al. Disruption of DDX53 coding sequence has limited impact on iPSC-derived human NGN2 neurons. BMC Med Genom. 2023;16:5. 10.1186/s12920-022-01425-3 PMC9837974 36635662

[61] Montani C, Gritti L, Beretta S, Verpelli C, Sala C. The synaptic and neuronal functions of the X-linked intellectual disability protein Interleukin-1 Receptor Accessory Protein Like 1 (IL1RAPL1). Dev Neurobiol. 2019;79:85–95. 30548231 10.1002/dneu.22657

[62] Kang HJ, Kawasawa YI, Cheng F, Zhu Y, Xu X, Li M, et al. Spatio-temporal transcriptome of the human brain. Nature. 2011;478:483–9. 22031440 10.1038/nature10523 PMC3566780

[63] Ramanan VK, Risacher SL, Nho K, Kim S, Shen L, McDonald BC, et al. GWAS of longitudinal amyloid accumulation on 18F-florbetapir PET in Alzheimer’s disease implicates microglial activation gene IL1RAP. Brain. 2015;138:3076–88. 26268530 10.1093/brain/awv231 PMC4671479

[64] Jiang Y, Zhou X, Wong HY, Ouyang L, Ip FCF, Chau VMN, et al. An IL1RL1 genetic variant lowers soluble ST2 levels and the risk effects of APOE-epsilon4 in female patients with Alzheimer’s disease. Nat Aging. 2022;2:616–34. 37117777 10.1038/s43587-022-00241-9 PMC10154240

[65] Callaerts-Vegh Z, Ahmed T, Vermaercke B, Marynen P, Balschun D, Froyen G, et al. Nxf7 deficiency impairs social exploration and spatio-cognitive abilities as well as hippocampal synaptic plasticity in mice. Front Behav Neurosci. 2015;9:179. 26217206 10.3389/fnbeh.2015.00179 PMC4498129

[66] Jun L, Frints S, Duhamel H, Herold A, Abad-Rodrigues J, Dotti C, et al. NXF5, a novel member of the nuclear RNA export factor family, is lost in a male patient with a syndromic form of mental retardation. Curr Biol. 2001;11:1381–91. 11566096 10.1016/S0960-9822(01)00419-5

[67] Lui JC, Wagner J, Zhou E, Dong L, Barnes KM, Jee YH, et al. Loss-of-function variant in SPIN4 causes an X-linked overgrowth syndrome. JCI Insight. 2023;8:e167074. 36927955 10.1172/jci.insight.167074 PMC10243798

[68] Luningham JM, Chen J, Tang S, De Jager PL, Bennett DA, Buchman AS, et al. Bayesian genome-wide TWAS method to leverage both cis- and trans-eQTL information through summary statistics. Am J Hum Genet. 2020;107:714–26. 32961112 10.1016/j.ajhg.2020.08.022 PMC7536614

[69] Bodily PM, Fujimoto MS, Page JT, Clement MJ, Ebbert MT, Ridge PG, et al. A novel approach for multi-SNP GWAS and its application in Alzheimer’s disease. BMC Bioinform. 2016;17:268. 10.1186/s12859-016-1093-7 PMC4965706 27453991

[70] Lam V, Takechi R, Pallebage-Gamarallage MM, Galloway S, Mamo JC. Colocalisation of plasma derived apo B lipoproteins with cerebral proteoglycans in a transgenic-amyloid model of Alzheimer’s disease. Neurosci Lett. 2011;492:160–4. 21310214 10.1016/j.neulet.2011.02.001

[71] Babelova A, Moreth K, Tsalastra-Greul W, Zeng-Brouwers J, Eickelberg O, Young MF, et al. Biglycan, a danger signal that activates the NLRP3 inflammasome via toll-like and P2X receptors. J Biol Chem. 2009;284:24035–48. 19605353 10.1074/jbc.M109.014266 PMC2781998

[72] Ying Z, Byun HR, Meng Q, Noble E, Zhang G, Yang X, et al. Biglycan gene connects metabolic dysfunction with brain disorder. Biochim Biophys Acta Mol Basis Dis. 2018;1864:3679–87. 30291886 10.1016/j.bbadis.2018.10.002 PMC6239930

[73] Li K, Ran B, Wang Y, Liu L, Li W. PLCgamma2 impacts microglia-related effectors revealing variants and pathways important in Alzheimer’s disease. Front Cell Dev Biol. 2022;10:999061. 36147734 10.3389/fcell.2022.999061 PMC9485805

[74] Hu L, Zang MD, Wang HX, Li JF, Su LP, Yan M, et al. Biglycan stimulates VEGF expression in endothelial cells by activating the TLR signaling pathway. Mol Oncol. 2016;10:1473–84. 27590684 10.1016/j.molonc.2016.08.002 PMC5423211

[75] Sims R, van der Lee SJ, Naj AC, Bellenguez C, Badarinarayan N, Jakobsdottir J, et al. Rare coding variants in PLCG2, ABI3, and TREM2 implicate microglial-mediated innate immunity in Alzheimer’s disease. Nat Genet. 2017;49:1373. 28714976 10.1038/ng.3916 PMC5669039

[76] Magno L, Bunney TD, Mead E, Svensson F, Bictash MN. TREM2/PLCgamma2 signalling in immune cells: function, structural insight, and potential therapeutic modulation. Mol Neurodegener. 2021;16:22. 33823896 10.1186/s13024-021-00436-5 PMC8022522

