# Supplementary material for: Chromosome X-wide association study in case control studies of pathologically confirmed Alzheimer’s disease in a European population
Source: Transl Psychiatry. 2024 Sep 4;14:358. doi: 10.1038/s41398-024-03058-9 (PMC11375158; doi:10.1038/s41398-024-03058-9)
Supplement: Supplementary file 1 — Supplementary Material [file 41398_2024_3058_MOESM1_ESM.docx]

## Supplementary Material


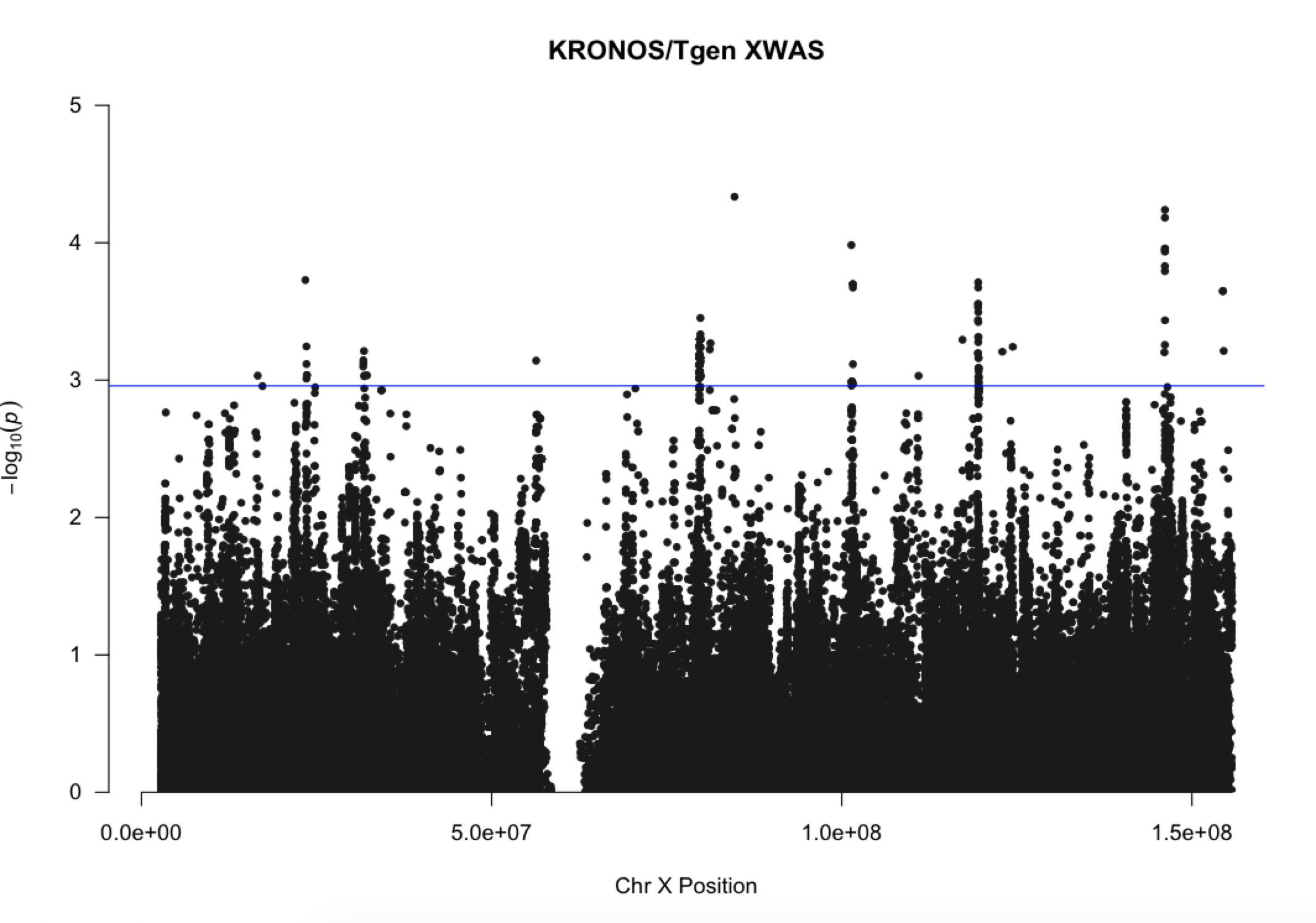


Supplementary Figure 1- Manhattan Plot of KRONOS/Tgen XWAS

Supplementary Table 1 - Top SNPs from KRONOS/Tgen XWAS Manhattan peaks

| **SNP** | **BP** | **OR** | | | **P** | **Nearest Gene** |
| --- | --- | --- | --- | --- | --- | --- |
|  |  | **Overall** | **Males** | **Females** |  |  |
| rs5923079 | 84682975 | 0.55 | 0.60 | 0.48 | 4.6e-05 | *HDX* |
| rs4827693 | 146138708 | 1.33 | 1.25 | 1.51 | 5.8e-05 | *TMEM257* |
| rs12848641 | 101367905 | 0.69 | 0.79 | 0.54 | 1.0e-04 | *BTK* |
| rs5910591 | 119469587 | 0.70 | 0.82 | 0.48 | 1.9e-04 | *SLC25A5* |
| rs186553004 | 81250064 | 0.35 | 0.47 | 0.26 | 5.4e-04 | *SH3BGRL* |
| rs5913102 | 79809325 | 0.74 | 0.74 | 0.74 | 3.5e-04 | *TBX22* |


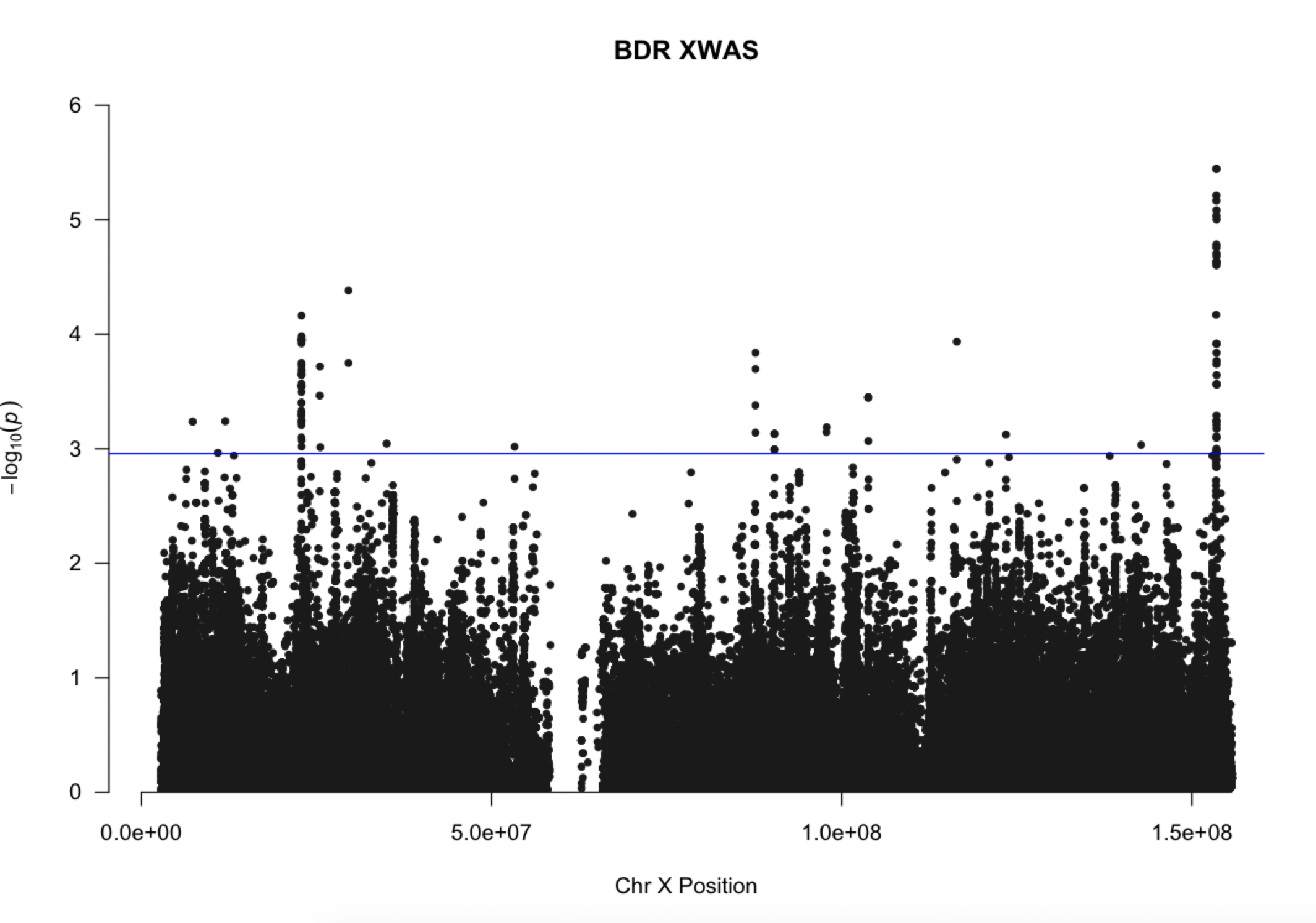


Supplementary Figure 2- Manhattan Plot of BDR XWAS

Supplementary Table 2- Top SNPs from BDR XWAS Manhattan peaks

| **SNP** | **BP** | **OR** | | | **P** | **Nearest Gene** |
| --- | --- | --- | --- | --- | --- | --- |
|  |  | **Overall** | **Males** | **Females** |  |  |
| rs2089596385 | 153481028 | 1.86 | 1.84 | 1.89 | 3.6e-06 | *HAUS7* |
| rs6628450 | 29551448 | 0.36 | 0.35 | 0.38 | 4.1e-05 | *IL1RAPL1* |
| rs12006935 | 22857207 | 0.52 | 0.48 | 0.62 | 6.9e-05 | *DDX53* |
| rs6520233 | 116420052 | 0.55 | 0.47 | 0.84 | 1.2e-04 | *SLC6A14* |
| rs72634525 | 87682263 | 0.46 | 0.50 | 0.40 | 1.5e-04 | *KLHL4* |


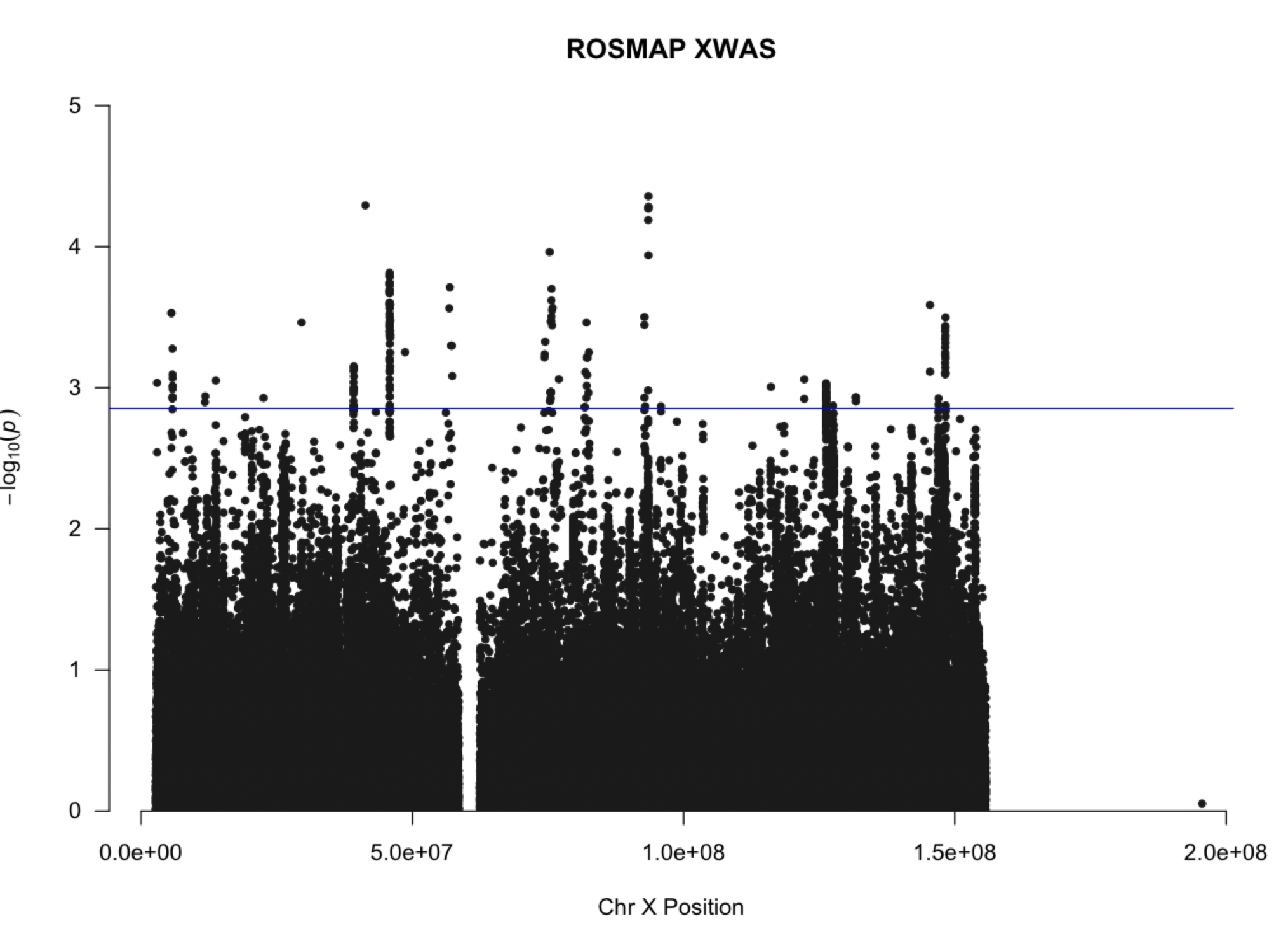


Supplementary Figure 3- Manhattan Plot of ROSMAP/MAYO/MSBB XWAS

Supplementary Table 3 - Top SNPs from ROSMAP/MAYO/MSBB XWAS Manhattan peaks

| **SNP** | **BP** | **OR** | | | **P** | **Nearest Gene** |
| --- | --- | --- | --- | --- | --- | --- |
|  |  | **Overall** | **Males** | **Females** |  |  |
| rs9969903 | 93503072 | 0.73 | 0.74 | 0.73 | 4.4e-05 | *NAPIL3* |
| rs112073726 | 75303891 | 0.55 | 0.39 | 0.67 | 1.1e-04 | *UPRT* |
| rs5906085 | 45823151 | 0.75 | 0.69 | 0.80 | 1.5e-04 | *CXorf36* |
| rs149966220 | 56914750 | 0.31 | 0.41 | 0.28 | 1.9e-04 | *SPIN3* |
| rs147450445 | 145399999 | 0.51 | 0.49 | 0.52 | 2.6e-04 | *SPANXN1* |
| rs137983810 | 29572683 | 0.52 | 0.51 | 0.52 | 3.5e-04 | *IL1RAPL1* |
| rs113157993 | 82089923 | 0.52 | 0.62 | 0.43 | 3.5e-04 | *SH3BGRL* |


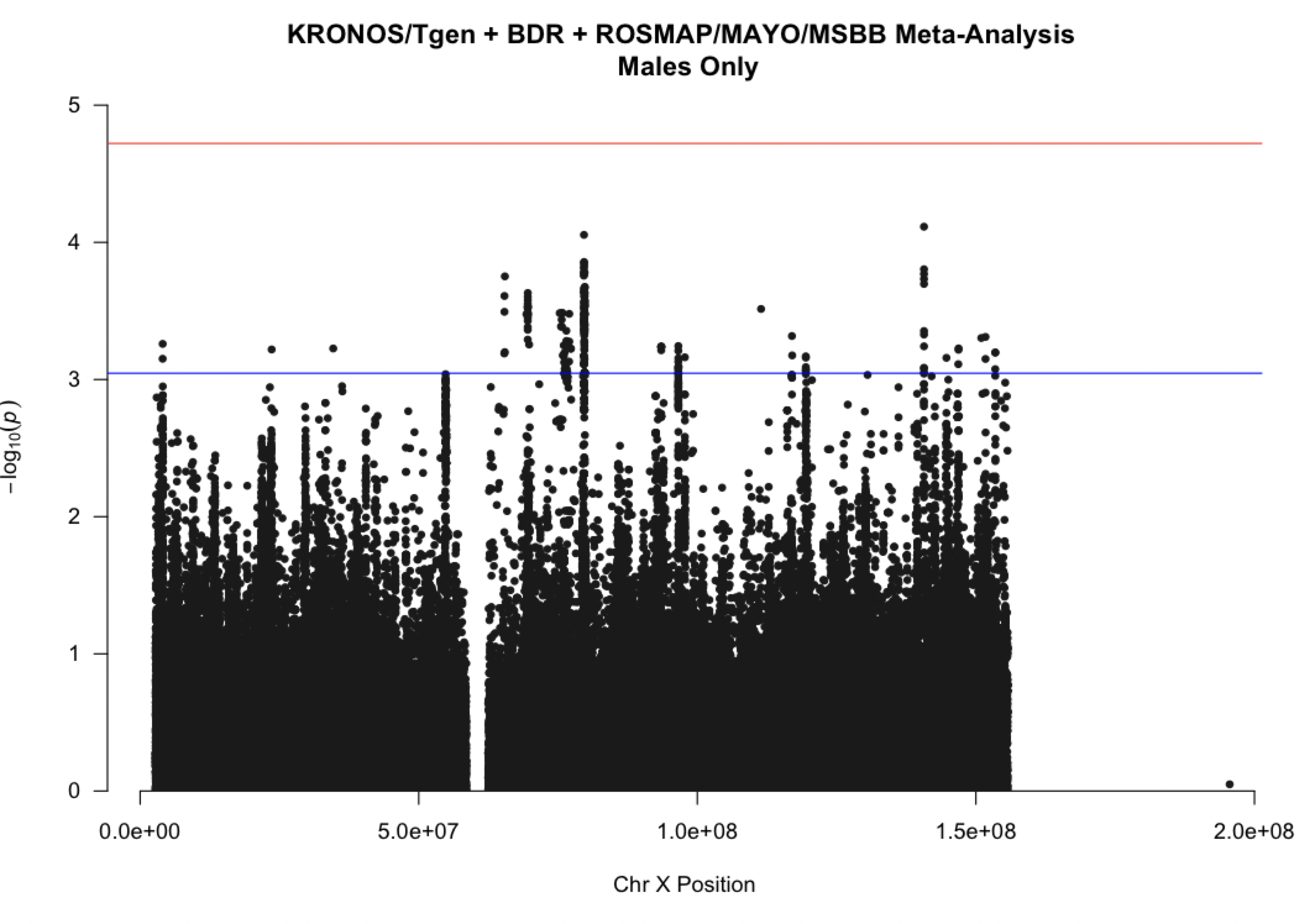


Supplementary Figure 4- Manhattan Plot of KRONOS/Tgen + BDR + ROSMAP/MAYO/MSBB XWAS in Males only


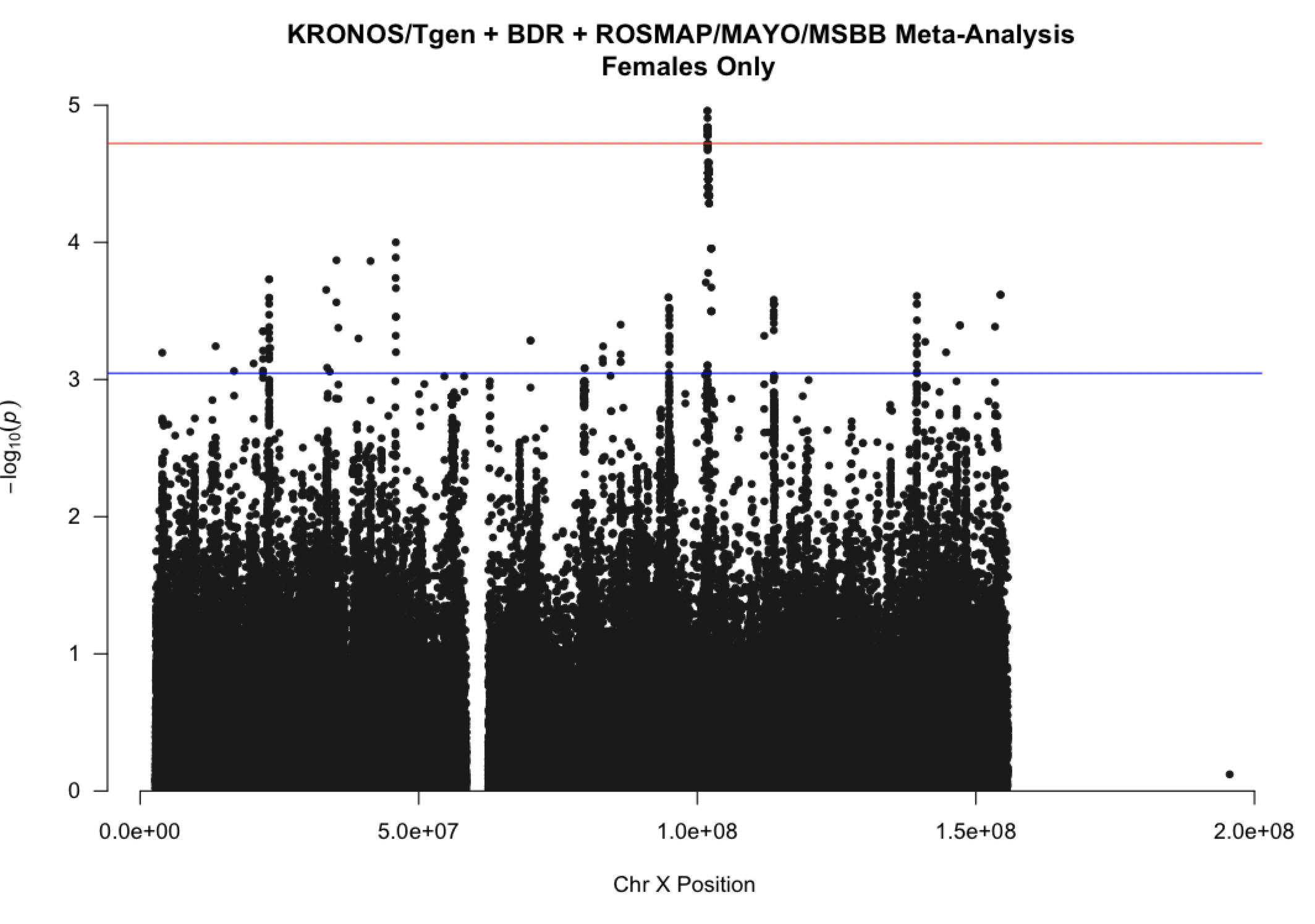


Supplementary Figure 5- Manhattan Plot of KRONOS/Tgen + BDR + ROSMAP/MAYO/MSBB XWAS in Females only

Supplementary Table 4 - Replicating SNPs from Meta-Analysis XWAS (KRONOS/Tgen+BDR+ROSMAP/MAYO/MSBB)

| **SNP** | **BP** | **OR** | | | **P** | **Nearest Gene** |
| --- | --- | --- | --- | --- | --- | --- |
|  |  | **Overall** | **Males** | **Females** |  |  |
| rs4827693 | 146138708 | 1.12 | 1.06 | 1.23 | 0.018 | *TMEM257* |
| rs12848641 | 101367905 | 0.76 | 0.86 | 0.64 | 0.002 | *BTK* |
| rs186553004 | 81250064 | 0.36 | 0.39 | 0.33 | 5.2e-05 | *SH3BGRL* |
| rs5913102 | 79809325 | 0.75 | 0.76 | 0.73 | 6.3e-07 | *TBX22* |
| rs2089596385 | 153481028 | 1.85 | 1.85 | 1.89 | 3.8e-06 | *HAUS7* |
| rs12006935 | 22857207 | 0.79 | 0.83 | 0.72 | 0.0005 | *DDX53* |
| rs9969903 | 93503072 | 0.88 | 0.89 | 0.86 | 0.0057 | *NAPIL3* |
| rs112073726 | 75303891 | 0.54 | 0.39 | 0.67 | 0.0002 | *UPRT* |
| rs147450445 | 145399999 | 0.68 | 0.69 | 0.68 | 0.002 | *SPANXN1* |
| rs137983810 | 29572683 | 0.77 | 0.77 | 0.75 | 0.038 | *IL1RAPL1* |


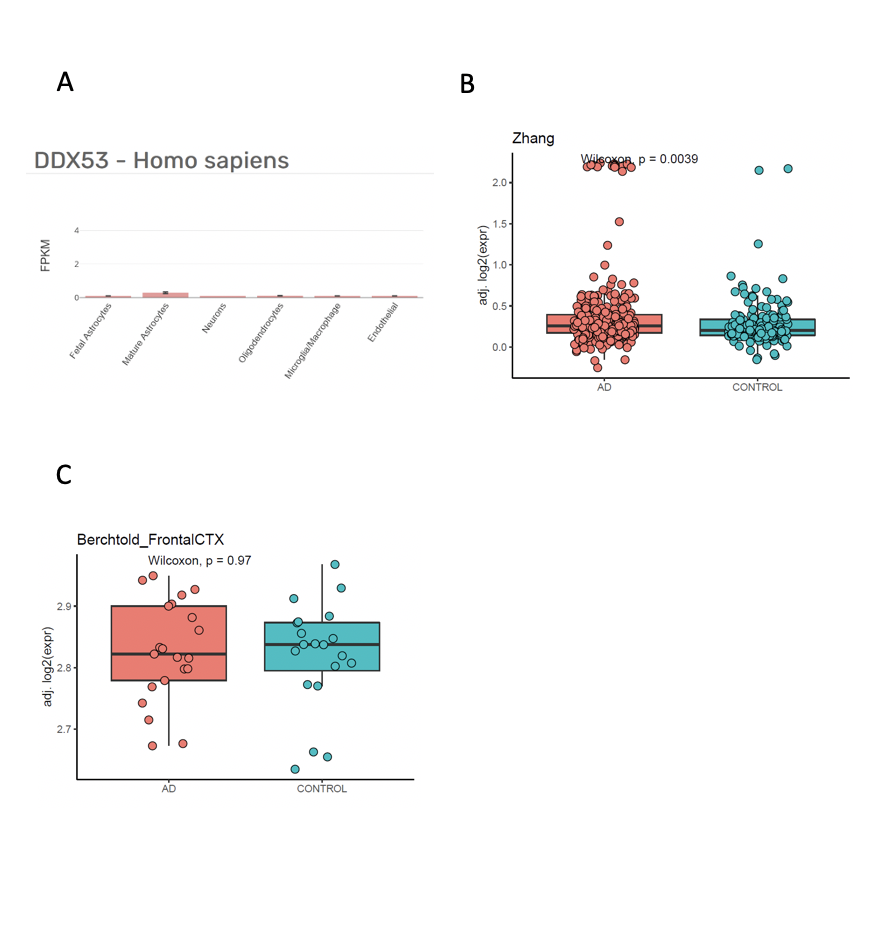


Supplementary Figure 6- Expression results for DDX53. A) Expression levels across several cell types, B) expression comparison in human AD cases compared to controls based on data from Zhang et al. (2013) and C) expression comparison in human AD cases compared to controls based on data from Berchtold et al. (2008).


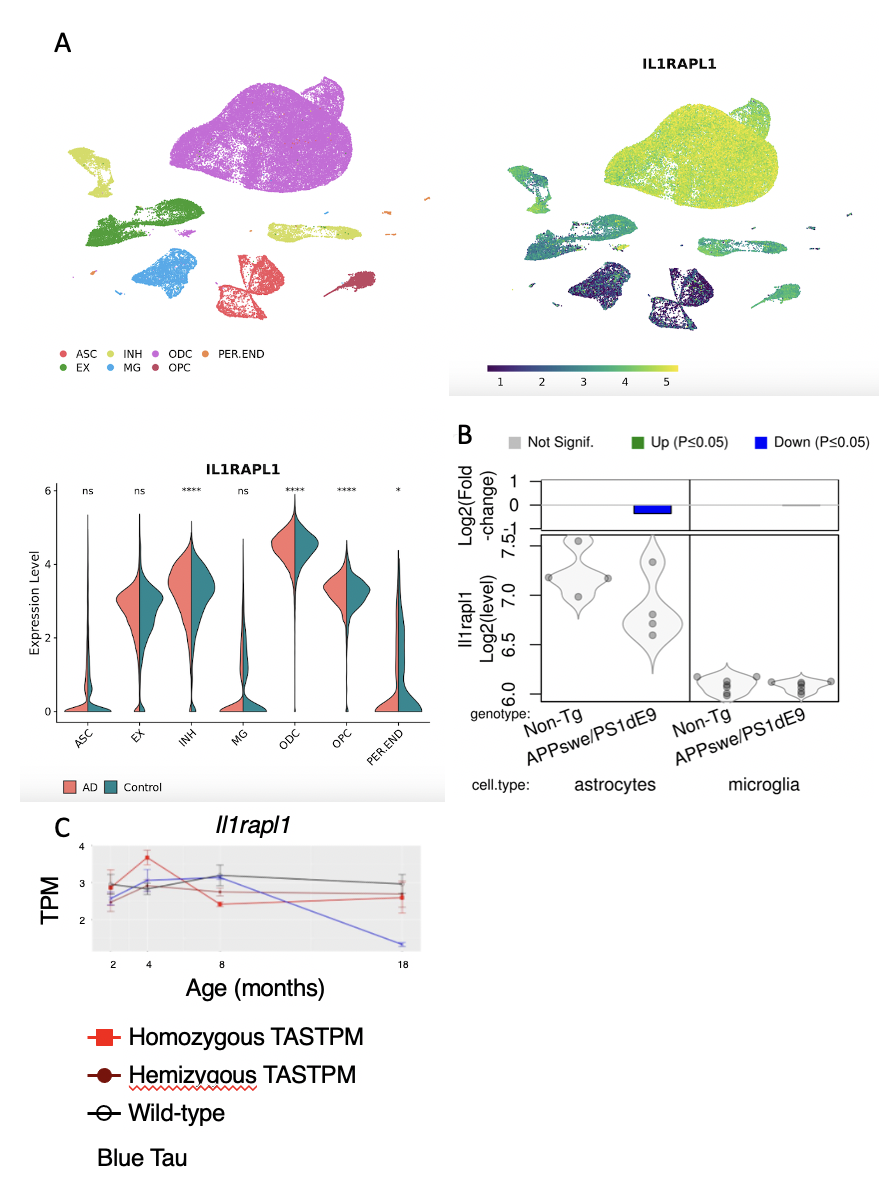


Supplementary Figure 7- Expression results for IL1RAPL1. A) Microglia expression levels across several human cell types, B) expression comparisons in mouse models of AD compared to age-matched wild-type controls in astrocytes and microglia and C) expression comparison in mouse models of AD compared to age-matched wild-type controls.

**
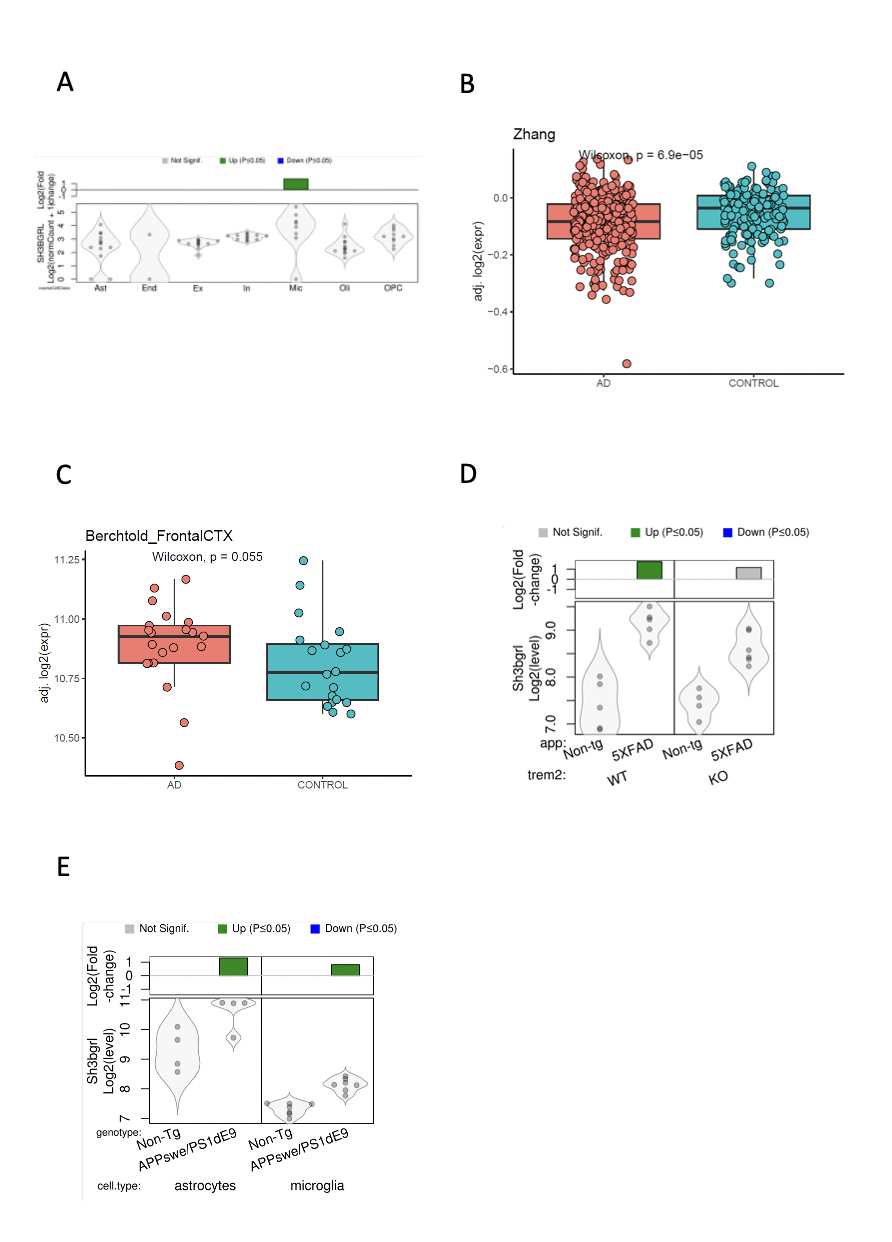
**

Supplementary Figure 8- Expression results for SH3BGRL. A) Human expression levels across several cell types, B) expression comparison in human AD cases compared to controls based on data from Zhang et al. (2013), C) expression comparison in human AD cases compared to controls based on data from Berchtold et al. (2008), D) expression comparisons in 5XFAD mouse model of amyloid pathology with and without Trem2 knockout compared to wild-type controls and E) expression comparisons in the APPswe/PSEN1dE9 mouse model of AD compared to age-matched wild-type controls in astrocytes and microglia.


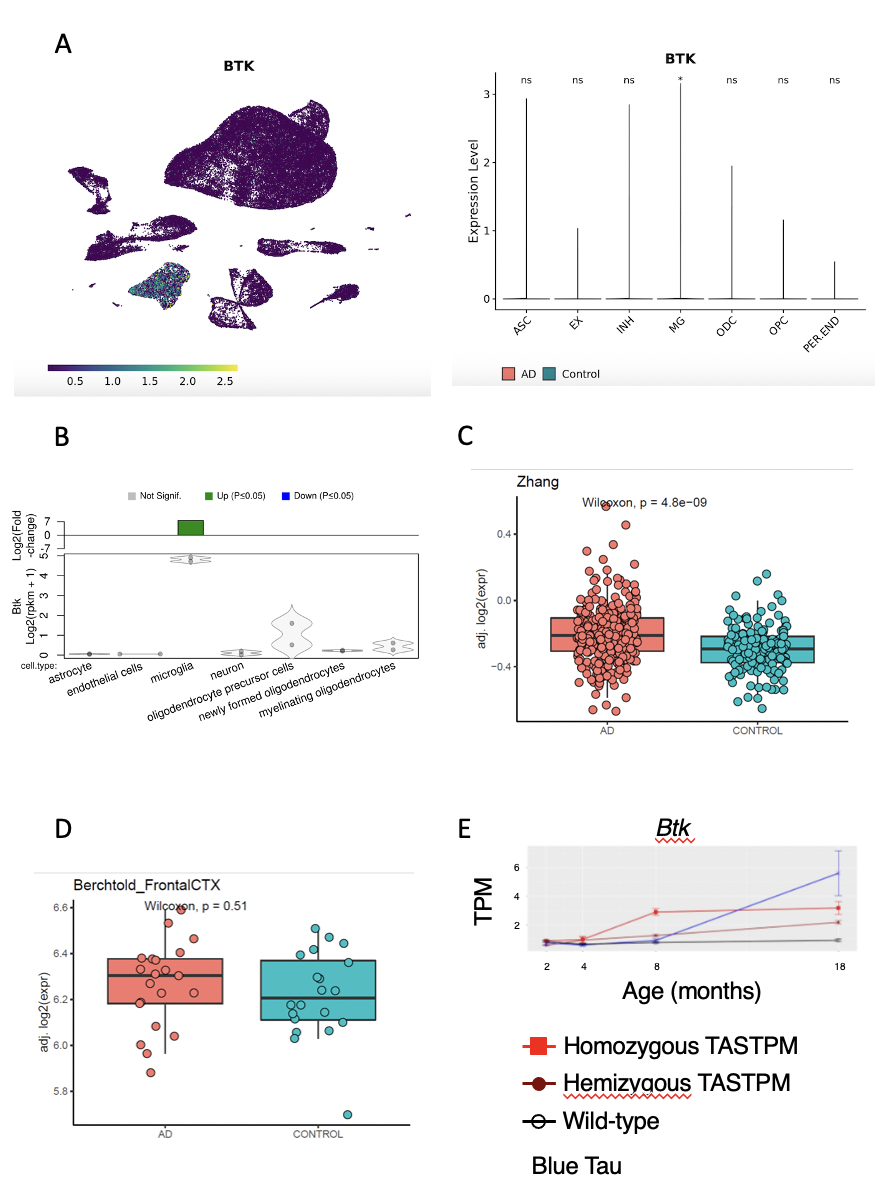


Supplementary Figure 9- Expression results for BTK. A) Microglia expression levels across several cell types, B) Human expression levels across several cell types, C) expression comparison in human AD cases compared to controls based on data from Zhang et al. (2013), D) expression comparison in human AD cases compared to controls based on data from Berchtold et al. (2008) E) expression comparison in mouse models of AD compared to age-matched wild-type controls.


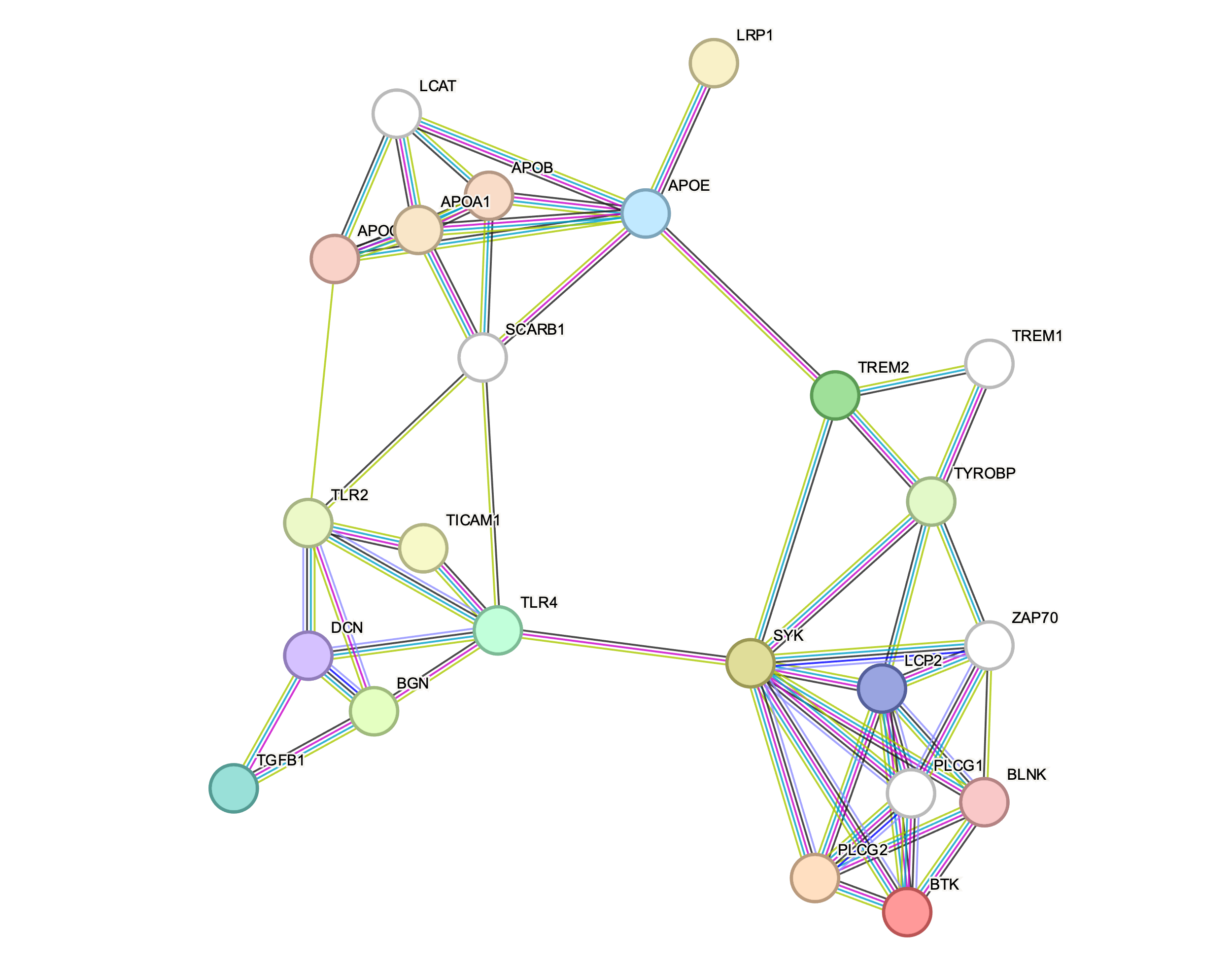


Supplementary Figure 10- BTK-based network from the STRING database. Solid lines indicate direct interactions (e.g. protein-protein interactions, or phosphorylation), and broken lines indirect interactions).

**
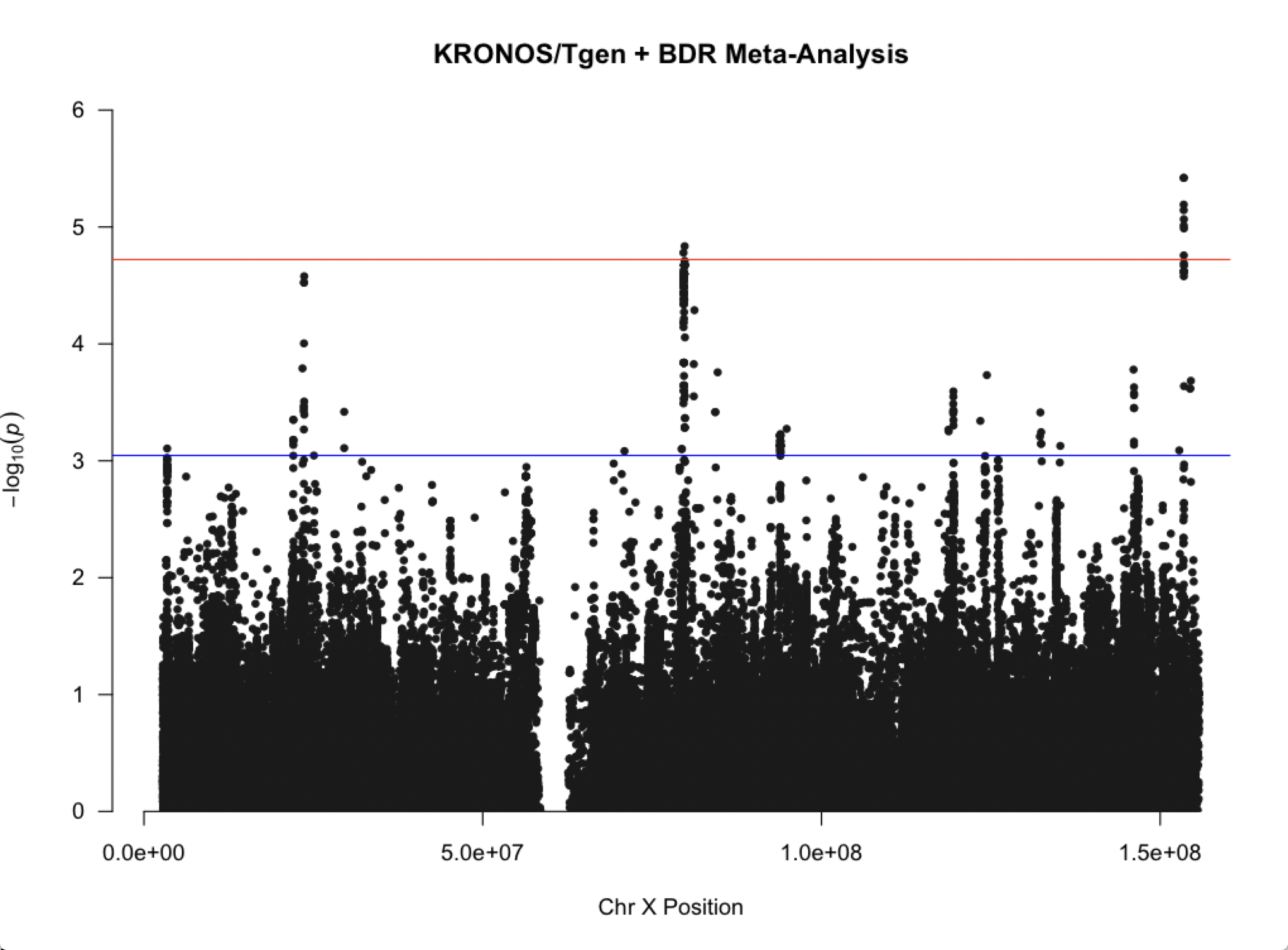
**

Supplementary Figure 11- Manhattan Plot of KRONOS/Tgen + BDR XWAS

Supplementary Table 5- Top SNPs from KRONOS/Tgen + BDR XWAS Manhattan peaks

| **SNP** | **BP** | **OR** | | | **P** | **Nearest Gene** |
| --- | --- | --- | --- | --- | --- | --- |
|  |  | **Overall** | **Males** | **Females** |  |  |
| rs2089596385 | 153481028 | 0.54 | 0.54 | 0.53 | 3.8e-06 | *HAUS7* |
| rs5913102 | 79809325 | 1.37 | 1.36 | 1.41 | 1.5e-05 | *TBX22* |
